# Quantitative pancreatic MRI in diabetes mellitus: current advances and future directions

**DOI:** 10.3389/fendo.2026.1887007

**Published:** 2026-06-29

**Authors:** Wenqi He, Chun Cao, Ping Jiang

**Affiliations:** 1 Department of Radiology, Zigong Fourth People’s Hospital, Sichuan, Zigong, China; 2 Department of Oncology, Zigong First People’s Hospital, Sichuan, Zigong, China

**Keywords:** artificial intelligence, MRI, pancreatic fat, quantitative imaging, type 1 diabetes, type 2 diabetes

## Abstract

Currently, the diagnosis and monitoring of diabetes mellitus (DM) primarily rely on clinical symptoms, blood glucose measurements, and laboratory blood tests. With the continuous advancement of medical imaging, magnetic resonance imaging (MRI) is increasingly being applied to the study of diabetes and its associated complications. Given that the pancreas plays a pivotal role in the pathogenesis and progression of DM, quantitative MRI techniques have emerged as powerful tools; they provide not only fundamental structural information but also visualize the pathophysiological alterations of the pancreas throughout the disease course. This literature review suggests that quantitative pancreatic MRI holds great promise as a novel, non-invasive biomarker for evaluating diabetic pathophysiology, facilitating early diagnosis, and monitoring therapeutic efficacy. Advancing this field further, the integration of artificial intelligence and radiomics offers powerful tools for automated high-throughput mining of these datasets, though its clinical translation remains preliminary. Current implementation gaps—most notably the critical need for multi-center external validation and standardized models—must be recognized before these deep imaging phenotypes can be reliably utilized in clinical settings.

## 1 Introduction

The global prevalence of diabetes mellitus (DM) is increasing at an alarming rate (1, 2), with type 2 diabetes mellitus (T2DM) (3) emerging as a formidable challenge to modern medicine and a critical threat to global public health. Traditionally, the diagnosis and longitudinal monitoring of DM have relied predominantly on clinical symptomatology, glycemic measurements, and standardized laboratory blood assays. Although magnetic resonance imaging (MRI) is routinely utilized for the diagnosis and staging of pancreatitis and pancreatic neoplasms (4–7), it was historically regarded as a redundant modality for DM management. This perspective was predicated on the conventional understanding that DM pathogenesis primarily involves the endocrine islets of Langerhans, which are too minute to be resolved via standard clinical MRI.

However, burgeoning evidence suggests that the exocrine pancreas also plays a pivotal role in the pathophysiology of DM. Quantitative MRI (qMRI) techniques provide not only fundamental morphometric data but also reveal underlying pathophysiological alterations associated with disease progression (8, 9). Furthermore, the standardized and reproducible nature of qMRI parameters facilitates multi-center clinical trials and longitudinal assessments, enabling cross-validation against histopathological ‘gold standards’ to eventually inform clinical guidelines. Beyond conventional anatomical sequences, qMRI encompasses a versatile suite of modalities, including chemical shift imaging (CSI), diffusion-weighted imaging (DWI), dynamic contrast-enhanced MRI (DCE-MRI), arterial spin labeling (ASL), and magnetic resonance elastography (MRE) (10). Specifically, CSI allows for the precise quantification of pancreatic fat fraction (11, 12); DWI characterizes cellular density and membrane integrity by evaluating the Brownian motion of water molecules (13); perfusion modalities such as DCE-MRI and ASL quantify microvascular density and hemodynamic fluctuations (14, 15); and MRE provides an assessment of parenchymal fibrosis by measuring tissue stiffness (16).

While prior reviews in this domain have predominantly concentrated on isolated morphological changes or solitary parameters like intra-pancreatic fat deposition, the unique novelty of this article lies in its systematic synthesis of recent peer-reviewed literature (2015–2026) to provide a holistic integration of these structural, diffusion (IVIM/DKI), perfusion dynamics (both DCE-MRI and ASL), and visco-elastic biophysical properties. By critically contrasting these multi-parametric phenotypes across both type 1 diabetes mellitus(T1DM) and T2DM etiologies and evaluating current artificial intelligence workflows alongside inter-vendor technical limitations, this review aims to bridge the gap between speculative research settings and standardized clinical practice.

## 2 Anatomical imaging

Historically, pancreatic volume (PV) was predominantly quantified using computed tomography (CT). However, the clinical utility of CT in pediatric and pregnant populations is constrained by the risks associated with ionizing radiation (17). Magnetic resonance imaging (MRI) has emerged as a superior non-ionizing alternative, offering exceptional soft-tissue contrast that facilitates precise anatomical delineation of the retroperitoneal pancreas (7). In clinical research, PV is typically derived from manual segmentation of pancreatic boundaries across contiguous axial MRI slices, followed by three-dimensional (3D) reconstruction and volumetric analysis using specialized visualization software (18).

**Figure 1:**
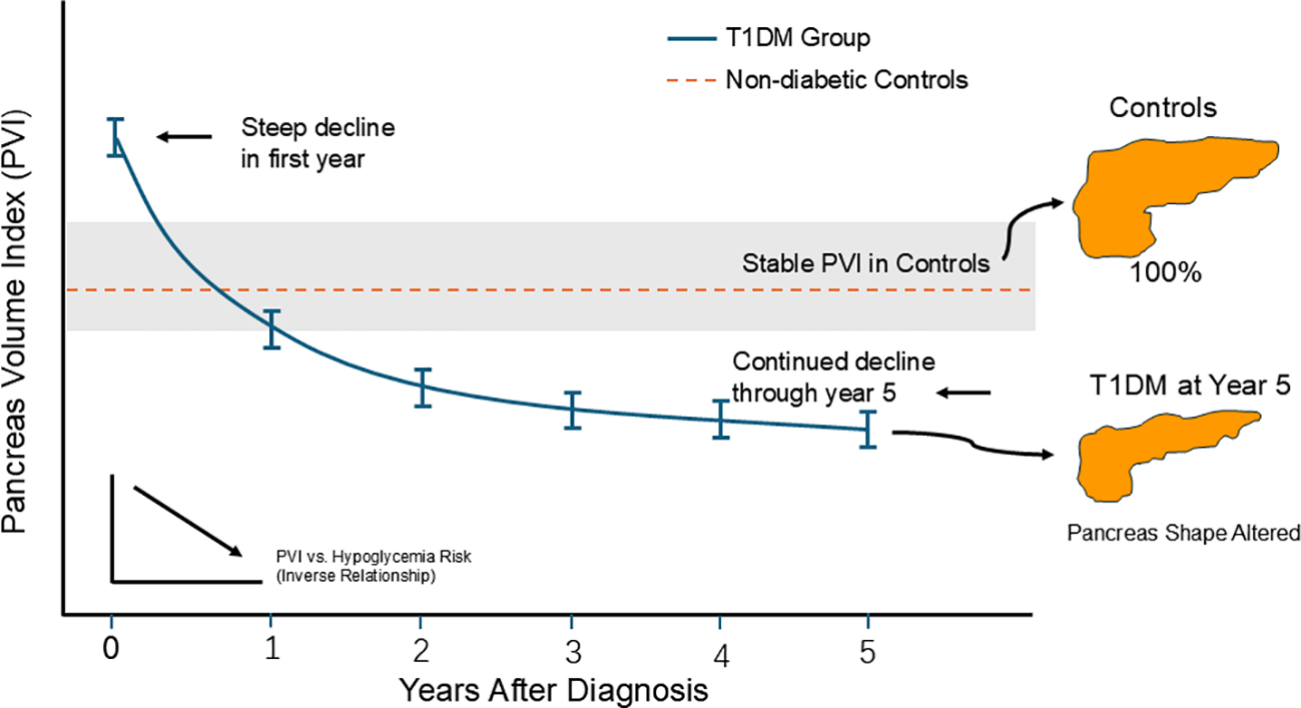
Longitudinal decline in pancreas volume index (PVI) after T1DM diagnosis. Longitudinal decline in Pancreas Volume Index (PVI) after T1DM diagnosis. The first year post-diagnosis shows a steep decline in PVI, followed by a continuous reduction up to year 5, accompanied by alterations in pancreatic morphology. There is an inverse correlation between PVI and the Low Blood Glucose Index, indicating an increased risk for hypoglycemia as PVI decreases. Figure generated independently by the authors based on numerical data from (19).

Recent longitudinal studies (19, 20) have demonstrated that patients with T1DM exhibit a rapid decline in both PV and the pancreatic volume index (PVI) within the first year of diagnosis, often coinciding with systemic weight loss. Notably, PVI continues to diminish over the subsequent five years, accompanied by progressive morphological distortions (**Figure 1**). Although the core pathogenesis of T1DM is the autoimmune destruction of pancreatic β-cells leading to absolute insulin deficiency, the endocrine islets constitute only 1%–2% of the total pancreatic parenchyma—a fraction insufficient to account for the observed degree of global pancreatic atrophy. Consequently, some investigators propose (21) that T1DM development is intrinsically linked to exocrine pancreatic insufficiency (EPI). Histological signatures of EPI include exocrine fibrosis, arteriosclerosis, fatty infiltration, and acinar atrophy. MRI-based assessments have confirmed that individuals at risk for EPI manifest significantly reduced PV, with progressive volume loss observed throughout the clinical trajectory of T1DM (19). These findings suggest that PV alterations may serve as a potent early biomarker for T1DM susceptibility and disease progression. Furthermore, reduced circulating levels of insulin-like growth factor 1 (IGF-1) and mutations in the insulin gene have been implicated as potential drivers of pancreatic atrophy in T1DM (22, 23), though these hypotheses necessitate further validation through large-scale, multicenter prospective trials.

Compared to T1DM, the relationship between T2DM and PV remains relatively underexplored. It remains elusive whether a reduced PV represents a predisposing factor for T2DM or a secondary sequela of chronic metabolic dysregulation. Japanese cohorts have characterized diminished PV and ‘serrated’ pancreatic margins as pathognomonic morphological features of T2DM (24). A landmark 2020 study published in *The Lancet* (25) utilized longitudinal MRI to monitor PV over a two-year period, revealing that patients who achieved remission of postprandial insulin secretion exhibited a significant restitution of PV (reaching 89% of healthy control values) and a marked reduction in morphological irregularities. These findings imply that pancreatic atrophy and structural disorganization in T2DM may be reversible consequences of the disease state rather than immutable inciting factors. This suggests that T2DM is a potentially modifiable condition in which pancreatic morphology can partially recover following successful metabolic intervention. Accordingly, MRI-based monitoring of pancreatic architecture holds significant promise as a non-invasive surrogate marker for assessing prognosis and therapeutic efficacy in T2DM.

Furthermore, studies on islet transplantation (26) have revealed a direct correlation between pancreatic dimensions and total islet count, which is further modulated by donor glycated hemoglobin (HbA1c) levels. This suggests that PV may serve as an integrated surrogate for both islet mass and secretory capacity. In healthy populations, PV undergoes dynamic transitions across the lifespan—expanding rapidly during childhood and undergoing senescent atrophy in later decades. While these fluctuations are primarily attributed to variations in body weight or body surface area (BSA), the specific role of diabetes mellitus in modulating these physiological processes warrants further investigation. Given its high precision and reproducibility in volumetric quantification, MRI is poised to be an indispensable tool in future diabetic research.

## 3 Chemical shift imaging

While anatomical imaging provides critical insights into macroscopic volume alterations, it cannot resolve the microscopic tissue composition changes that drive pancreatic atrophy. Beyond global structural loss, when ectopic lipid deposition occurs when the systemic influx of free fatty acids exceeds the buffering capacity of adipose tissue, leading to deleterious accumulation within non-adipose viscera, including the pancreas, liver, and skeletal muscle (27, 28). Elevated intra-pancreatic fat deposition (IPFD) is strongly associated with an increased risk of acute pancreatitis, pancreatic adenocarcinoma, and diabetes mellitus (29). Within the pancreatic microenvironment, complex paracrine crosstalk involving adipocytes, proinflammatory immune cells, and β-cells may precipitate β-cell exhaustion and apoptosis, potentially serving as a primary driver in the pathogenesis of T2DM (30, 31).

Quantitative MRI facilitates the non-invasive assessment of IPFD by exploiting the differential resonance frequencies of protons in water and lipid molecules. Historically, magnetic resonance spectroscopy (MRS) was regarded as the ‘gold standard’ for lipid quantification due to its high sensitivity (32); however, its application in pancreatic imaging is frequently compromised by respiratory motion artifacts and magnetic field inhomogeneities. These constraints increase the coefficient of variation in the pancreas compared to the liver, thereby limiting its diagnostic precision. In contrast, chemical shift imaging (CSI) offers superior anatomical localization and mitigates partial volume effects from peripancreatic fat, yielding more robust and reproducible IPFD measurements (33). Chai et al. (34) demonstrated that IPFD is significantly elevated in treatment-naïve T2DM patients compared to normoglycemic controls, suggesting that pancreatic steatosis is a pivotal risk factor. While Skudder-Hill et al. (35) observed no significant spatial heterogeneity in fat distribution across the pancreatic head, body, and tail, increased lipid accumulation specifically in the body and tail was positively correlated with exacerbated insulin resistance—highlighting its potential for early risk stratification.

**Figure 2:**
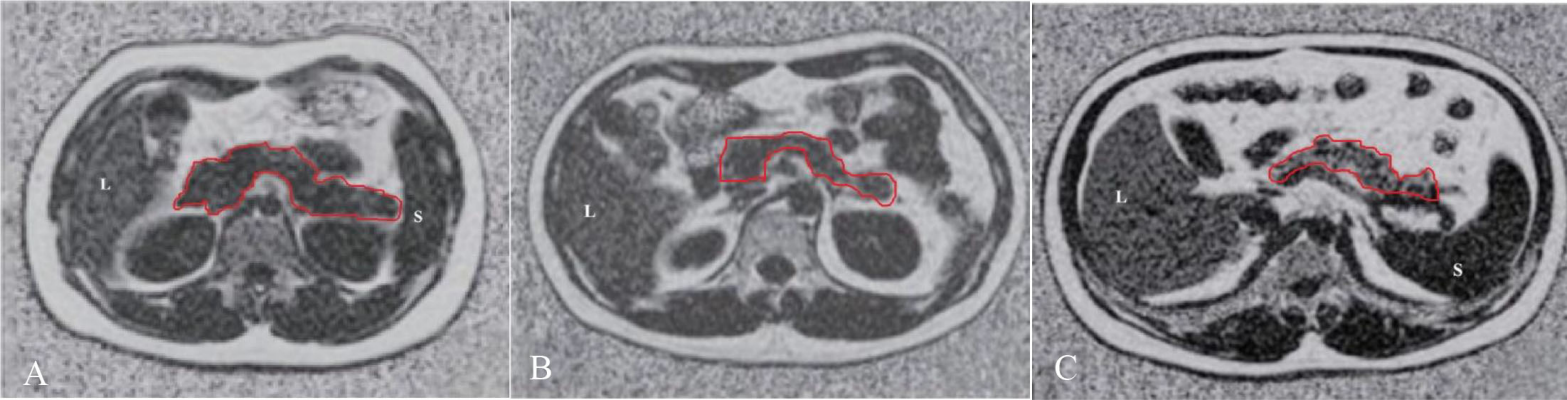
Representative quantitative pancreatic fat fraction mappings (Dixon MRI) across the glycemic continuum. Red contours delineate regions of interest (ROIs) manually segmented along anatomical parenchymal boundaries, strictly excluding peripancreatic adipose tissue. **(A)** Normoglycemic control (NGT) demonstrating uniform, healthy parenchyma. **(B)** Impaired glucose tolerance (IGT) showing intermediate lipid accumulation. **(C)** Newly diagnosed T2DM exhibiting severe, diffuse fatty infiltration. L, liver; S, spleen. Adapted and modified from Liang et al. (36), originally published in Journal of Sun Yat-sen University (Medical Sciences) under the terms of the Creative Commons Attribution-NonCommercial 4.0 International License (CC BY-NC 4.0).

Recent advancements, such as the Iterative Decomposition of Water and Fat with Echo Asymmetry and Least Square Estimation (IDEAL-IQ), have revealed a stepwise increment in pancreatic fat volume fraction (PFVF) across the glycemic continuum, peaking in manifest T2DM (36)(**Figure 2**). This gradient underscores the role of fatty infiltration in disease progression and suggests that MRI-derived fat quantification could serve as a sensitive biomarker for therapeutic monitoring. Similarly, 6-point Dixon MRI provides quantification accuracy comparable to MRS while offering superior spatial resolution and shorter acquisition times, making it increasingly favored for small-organ evaluation (37). Findings indicate that T2DM patients exhibit significantly higher fat fractions across all pancreatic segments compared to both pregnant and young healthy cohorts.

In contrast to T2DM, recent multiparametric imaging and genetic causal inference indicate that pancreatic steatosis is not a primary feature of T1DM. A multiparametric 3-T MRI study using proton density fat fraction (PDFF) mapping revealed that patients with long-standing T1DM exhibit low pancreatic fat content that does not significantly differ from healthy controls (2.2% vs 4.0%). Instead of fatty infiltration, the T1DM pancreas exhibits severe tissue atrophy and fibro-atrophic remodeling, characterized by prolonged T1 relaxation times and altered diffusion metrics (38). This phenotypic independence is substantiated at the genomic level by Mendelian randomization analyses, which demonstrate that genetically predicted pancreatic fat fraction bears no causal relationship with T1DM risk (39). Thus, while quantitative chemical shift imaging is highly sensitive for tracking the metabolic disruptions unique to T2DM, pancreatic adiposity is neither a causal driver nor a hallmark of autoimmune-mediated T1DM.

Nevertheless, the clinical utility of quantitative MRI in monitoring IPFD for the prognosis and resolution of gestational diabetes remains an area for future investigation. However, it is worth noting that the clinical translation of these findings remains constrained by certain methodological limitations. A significant proportion of current literature evaluating intra-pancreatic fat deposition (IPFD) via CSI or IDEAL-IQ is predominantly characterized by single-center experiences and retrospective designs with relatively modest sample sizes. Consequently, these preliminary observations lack robust validation from large-scale, multi-center prospective cohorts, which is essential to establish generalized diagnostic thresholds across diverse populations.

## 4 AI and radiomics

To address these methodological limitations—particularly the low throughput of manual segmentation and the subtlety of early pancreatic heterogeneities—the transition from operator-dependent manual workflows to artificial intelligence (AI)-integrated networks represents a paradigm shift in IPFD assessment. AI-driven automation enhances the stability and throughput of volumetric and fat-fraction analysis, facilitating the generation of robust, large-scale evidence (40). To achieve automated high-throughput analysis, convolutional neural networks (CNNs)—including multi-stage networks integrated with decision fusion for MRI scans (41)—and particularly standard U-Net architectures, are widely employed for pancreatic boundary definition (42, 43). Recent deep learning advancements have introduced variants to overcome anatomical variability and poorly defined borders; for instance, V-Net optimizes 3D volumetric spatial features (44), while attention-gated Transformers enhance accuracy by capturing long-range contextual dependencies (45, 46). These architectural evolutions ensure robust, reproducible region-of-interest (ROI) segmentation, establishing a critical foundation for downstream radiomics feature extraction.

Notably, a deep learning radiomics (DLR) model was recently developed to quantify the proton density fat fraction (PDFF) via multi-echo Dixon imaging. This model achieved an area under the curve (AUC) of 0.868 in differentiating between individuals with T2DM, pre-diabetes, and those with normal glucose tolerance, notably outperforming experienced radiologists, who yielded AUCs of 0.760 and 0.782 (47). Such technological advancements offer a more efficient and standardized framework for risk stratification and clinical decision-making, establishing IPFD as a potent early biomarker for T2DM.

Building upon these advancements, integrating radiomics significantly expands the clinical utility of quantitative pancreatic MRI by accurately quantifying islet and acinar structures, predicting disease progression, and segmenting sub-visual features (48, 49). Crucially, radiomics leverages high-throughput computing to extract hundreds of distinct texture features from conventional images, thereby mapping the microstructural and spatial heterogeneities of the pancreatic parenchyma induced by chronic inflammation, early-stage fibrosis, or diffuse micro-fat deposition (50, 51). These computational artificial intelligence (AI) frameworks can decipher subtle tissue alterations that escape conventional qualitative radiological inspection, offering an objective pipeline to forecast glycemic deterioration and monitor early therapeutic responses.

However, routine clinical translation remains distinctly preliminary. A paramount bottleneck is the systemic lack of standardized validation criteria; most published models suffer from potential overfitting and a critical deficit in testing against independent, multi-center external validation cohorts. To bridge the gap between speculative algorithmic capability and reproducible clinical practice, future research must strictly adhere to established reporting guidelines, such as the Transparent Reporting of a multivariable prediction model for Individual Prognosis or diagnosis (TRIPOD) statement and the clinical evaluation of artificial intelligence radiology publications (CLEAR) checklist. Establishing open-source benchmark datasets and conducting multi-vendor prospective trials are mandatory prerequisites to ensure algorithmic safety and model generalizability before these deep imaging phenotypes can be reliably deployed for active clinical decision-making.

## 5 Diffusion-weighted imaging

In addition to mapping tissue fat and morphology via standard sequences and intelligent radiomics, capturing the microscopic motion of water molecules provides another crucial dimension for assessing pancreatic microenvironmental changes. Specifically, DWI provides non-invasive insights into the Brownian motion of water molecules within biological tissues, thereby reflecting cellular density and membrane integrity (52, 53). Quantification is achieved by modulating diffusion-sensitizing gradients (b-values) and evaluating signal attenuation to generate the apparent diffusion coefficient (ADC) (54). While historically rooted in neuroradiology (55, 56), advancements in MRI hardware and rapid acquisition techniques have expanded the clinical utility of DWI to the diagnosis of pancreatic neoplasms and inflammatory disorders (57–59).

Currently, research investigating DWI-based pancreatic alterations in diabetes remains nascent. One study observed that ADC values across all pancreatic segments were significantly lower in patients with fulminant T1DM compared to healthy controls, yielding a sensitivity of 86% and a specificity of 71% (60). Similar uniform reductions in ADC were noted in atypical fulminant T1DM, suggesting that restricted diffusion may encapsulate acute parenchymal changes during disease onset.

## 6 Intravoxel incoherent motion

As an advanced derivative of diffusion-weighted imaging (DWI), intravoxel incoherent motion (IVIM) utilizes multi-b-value acquisition coupled with a bi-exponential fitting model to delineate pure molecular diffusion from microvascular perfusion (61). This functional imaging approach has been successfully implemented to characterize pancreatic alterations across the glycemic spectrum, encompassing type 2 diabetes mellitus (T2DM), asymptomatic hyperglycemia, and normoglycemic controls. IVIM-DWI facilitates the extraction of pivotal quantitative metrics, including the true diffusion coefficient (D or Dt), the pseudo-diffusion coefficient (D* or Dp), and the perfusion fraction (f) (62).

Accumulating evidence indicates that these imaging-derived parameters correlate significantly with biochemical indices of β-cell function, such as the Composite Insulin Sensitivity Index (ISI), the 60-min Insulinogenic Index (IGI60), and the Disposition Index (DI). Notably, Dt has demonstrated superior diagnostic efficacy in discriminating asymptomatic hyperglycemic individuals from healthy volunteers. Furthermore, IVIM parameters exhibit robust correlations with composite ISI and DI, with Dt manifesting the strongest association with the DI. These findings underscore the potential of IVIM-DWI as a non-invasive quantitative surrogate for identifying early-stage pancreatic *β*-cell dysfunction, thereby offering a transformative diagnostic paradigm for the precision management of T2DM.

## 7 Diffusion kurtosis imaging

Diffusion kurtosis imaging (DKI) further enhances the diagnostic potential of diffusion MRI by accounting for the non-Gaussian diffusion of water molecules *in vivo*. By quantifying the degree of kurtosis, DKI characterizes the complexity of the tissue microenvironment with greater fidelity than conventional DWI. While DKI research in diabetes has primarily focused on neurostructural changes and diabetic nephropathy (63, 64), its application in the pancreas remains sparse. A Japanese cohort study (65) categorized 102 subjects based on HbA1c levels (<5.7%, 5.7%–6.5%, and >6.5%). Using DKI, researchers calculated the Mean Kurtosis (MK) of the pancreatic parenchyma and identified a significant positive correlation between MK and HbA1c levels. For the detection of patients with HbA1c >6.5%, MK demonstrated a sensitivity of 90%, a specificity of 88%, and an area under the ROC curve (AUC) of 0.92. This evidence suggests that pancreatic MK could serve as a robust imaging surrogate for chronic glycemic status. Given its high diagnostic accuracy, DKI-derived MK holds potential as a valuable, radiation-free tool for monitoring glycemic control, particularly in clinical contexts where HbA1c measurements may be confounded.

Despite these promising results, several methodological challenges hamper the clinical reproducibility of diffusion-derived metrics in pancreatic imaging. Owing to the organ’s small volume, deep retroperitoneal location, and high susceptibility to gastrointestinal peristalsis and respiratory motion artifacts, quantitative diffusion parameters remain highly volatile. These technical constraints often lead to substantial intra- and inter-study fluctuations in calculated ADC and D* values, thereby complicating the cross-validation of results and underlining the urgent need for standardized acquisition sequences.

## 8 Perfusion imaging

Beyond assessing the stationary diffusion of interstitial water molecules, evaluating the continuous dynamic flow of blood within the pancreatic microvasculature is equally essential. Since the endocrine pancreas is characterized by a dense, specialized capillary network, magnetic resonance perfusion imaging is a well-established modality for quantifying hemodynamics in the brain and myocardium (66–68); however, its application in pancreatic research remains relatively nascent (69). The endocrine pancreas is characterized by a dense, specialized capillary network. In both T1DM and T2DM, the blood flow rate within the endocrine islets is significantly higher than that in the surrounding exocrine parenchyma. As a non-invasive surrogate for assessing islet microcirculation, magnetic resonance perfusion imaging holds significant clinical promise (70). The following sections review the advancements in two distinct MRI perfusion techniques for the study of diabetes.

### 8.1 Dynamic contrast-enhanced MRI

DCE-MRI involves the serial acquisition of T1WI images following the intravenous administration of a paramagnetic contrast agent. The ‘dynamic’ utility of this technique arises from the continuous temporal tracking of contrast agent distribution throughout the tissue (71). The resulting voxel-wise time-intensity curves can be analyzed via pharmacokinetic modeling to estimate specific perfusion parameters. A study investigating the nexus between visceral adipose tissue (VAT), impaired pancreatic perfusion, and β-cell dysfunction in obese female patients with diabetes suggested that diminished pancreatic perfusion may not be directly linked to β-cell failure during the nascent stages of T2DM. However, perfusion levels in the pancreatic head were negatively correlated with both VAT and fasting plasma glucose (FPG) (72). This phenomenon may be attributed to the pancreatic head serving as a preferential site for ectopic fat deposition in this cohort. Excessive adiposity may exert mechanical compression on the microvasculature or disrupt the balance of angiogenic factors, thereby inhibiting neovascularization. Furthermore, impaired endothelial function may compromise vasoreactivity, further attenuating perfusion in the pancreatic head.

### 8.2 Arterial spin labeling

ASL is a non-invasive MRI modality that enables the quantification of tissue perfusion without the necessity of exogenous contrast media (73). By employing magnetically labeled arterial blood as an endogenous tracer, ASL effectively characterizes regional variations in pancreatic microvascular density. Evidence indicates that ASL findings are highly concordant with those derived from dynamic contrast-enhanced DCE-MRI; for instance, ASL-based assessments have demonstrated a systemic augmentation in pancreatic perfusion following a glucose challenge (74, 75).

Furthermore, ASL has been utilized to characterize postprandial pancreatic blood flow (PBF) dynamics across the glycemic spectrum. Longitudinal observations reveal that PBF is significantly diminished in T2DM and pre-diabetic cohorts across all temporal points compared to individuals with normal glucose tolerance (NGT). In all physiological groups, PBF typically reaches its peak 15 minutes post-ingestion before undergoing a gradual decline. Notably, the baseline PBF in the pancreatic tail (BL-PBF_tail_) has demonstrated superior diagnostic efficacy in identifying impaired glucose tolerance and has been identified as an independent risk factor for dysglycemia (76). These collective findings underscore the clinical utility of ASL in the non-invasive characterization of PBF hemodynamics and endocrine fluctuations, highlighting its potential for the early detection and precision management of diabetes.

Although human pancreatic perfusion studies in T1DM remain sparse, preclinical contrast-enhanced ultrasound (CEUS) utilizing microbubble destruction-replenishment imaging has demonstrated that microvascular perfusion dynamics—specifically an increased reperfusion rate and decreased amplitude—can reliably track insulitis and disease progression. Crucially, this blunting in pancreatic reperfusion rate successfully predicted therapeutic responsiveness to immunotherapies and β-cell-protective agents before disease onset (77, 78). Because these CEUS-derived kinetics closely mirror hemodynamic parameters accessible via advanced Perfusion MRI (such as DCE-MRI or ASL), these findings provide a vital translational foundation. Implementing corresponding quantitative MRI protocols could effectively extend these preclinical microvascular insights into clinical T1DM management, establishing non-invasive MR perfusion imaging as a predictive biomarker for therapeutic monitoring.

## 9 Magnetic resonance elastography

Long-term abnormalities in microvascular perfusion, combined with progressive steatosis, eventually culminate in chronic tissue inflammation and structural remodeling. To characterize the mechanical consequences of these pathological processes, MRE is a specialized imaging modality that quantifies the visco-elastic properties of biological tissues by detecting the propagation of shear waves generated by an external acoustic driver. In contrast to longitudinal waves, shear waves propagate with higher velocity and wavelength in stiffer tissues (79). Although the primary clinical utility of MRE has been the non-invasive staging of hepatic fibrosis and cirrhosis (80, 81), its application to pancreatic assessment has historically been hampered by the organ’s diminutive volume and deep retroperitoneal location.

Recent evidence, however, indicates that chronic pancreatitis and pancreatic neoplasms are characterized by significantly elevated parenchymal stiffness. Furthermore, dynamic shifts in pancreatic stiffness have been observed following oral glucose challenges in obese cohorts. Notably, patients with DM exhibit significantly higher pancreatic stiffness compared to healthy controls. MRE-derived stiffness values have demonstrated robust correlations with the histological severity of pancreatic fibrosis and acinar atrophy (82, 83). The increased stiffness observed in the diabetic pancreas is likely attributable to progressive fibrosis secondary to IPFD. The ectopic accumulation of adipocytes and macrophages within the pancreatic microenvironment triggers the secretion of pro-inflammatory cytokines, which perpetuate chronic inflammation and activate stellate cells, thereby driving fibrotic remodeling. Furthermore, a critical gap in current pancreatic MRE research is the profound lack of direct histopathological correlation. To date, the majority of clinical studies have merely performed correlation analyses between MRE-derived stiffness values and peripheral biochemical indices or clinical scores. Without direct cross-validation against histopathological ‘gold standards’ (such as biopsy or surgical specimens) to definitively map stiffness to specific stages of extracellular matrix deposition or acinar atrophy, the pathophysiological interpretation of pancreatic shear stiffness remains largely associative rather than causative. While pancreatic MRE has demonstrated high reproducibility in test-retest assessments, its diagnostic precision remains contingent upon the optimization of mechanical vibration frequencies and may be confounded by age-related physiological changes.

## 10 Sources of technical heterogeneity in quantitative pancreatic MRI

Extrapolating and comparing quantitative pancreatic MRI findings across multicenter longitudinal studies remains inherently challenging due to significant technical heterogeneity. Multiple confounding sources contribute to this inter-study variability:

Magnetic Field Strength (1.5T vs. 3.0T): Field strength fundamentally shifts the proton chemical shift frequency, altering water-fat separation performance in CSI. It also accelerates shear wave attenuation during MRE, causing divergent stiffness estimations (84, 85).

DWI Parameters: The selection and maximum value threshold of b-values drastically modify the fitting algorithms of the ADC and DKI mathematical models (86).

Vendor-Specific Solutions: Commercial reconstruction algorithms vary inherently among vendors; for example, GE’s IDEAL-IQ, Siemens’ LiverLab and Philips’ mDIXON-Quant platforms incorporate distinct spectral modeling for fat fraction maps, introducing systematic variations even under identical imaging protocols (87, 88). Such discrepancies fundamentally impair the longitudinal repeatability of multi-center trial data, proving that absolute diagnostic cut-offs cannot be uniformly applied without cross-vendor baseline harmonization.

To visually synthesize these parameters, the primary technical confounders and their explicit impacts are summarized in **Table 1**.

**Table 1:** Summary of technical parameters contributing to inter-study variability in quantitative pancreatic MRI.

Technical parameter/confounder	Modality/sequence context	Specific mechanism/clinical impact
Magnetic Field Strength (1.5T vs. 3.0T)	CSI&MRE	Shifts water-fat separation frequencies; accelerates shear wave attenuation
B-value Selection (Thresholds& distribution)	DWI&DKI	Alters mathematical model curve fitting, shifting baseline ADC and DKI metrics
Vendor-Specific Sequences(e.g., IDEAL-IQ vs. mDIXON-Quant vs. LiverLab)	Fat Quantification	Employs proprietary spectral modeling, leading to systemic measurement bias

ADC, apparent diffusion coefficient; CSI, chemical shift imaging; DKI, diffusion kurtosis imaging.

## 11 Conclusion and future perspectives

In conclusion, given the escalating global prevalence of DM and its deleterious complications, there is a pressing clinical exigency for advanced diagnostic and therapeutic monitoring tools. Accumulating evidence underscores the pivotal involvement of the pancreas throughout the clinical continuum of DM. As summarized in **Table 2**, multiparametric qMRI facilitates the non-invasive characterization of both macro- and microstructural alterations—encompassing pancreatic volume, parenchymal stiffness, fat fraction, microvascular perfusion, cellular density, and membrane integrity. These metrics provide indispensable biomarkers for the clinical diagnosis, phenotyping, and longitudinal monitoring of DM.

**Table 2:** Quantitative pancreatic MRI alterations in patients with diabetes mellitus.

MRI technique	Application/parameters	T1DM alterations	T2DM alterations	Biological confounders	Research limitations	Clinical preparedness level
Anatomical Imaging	Pancreatic Volume	Significant decrease	Decrease	Age, Weight	Partial volume effects; lack of multi-center standardization	**High** (Standard clinical sequence)
Chemical shift imaging	Pancreatic Fat Fraction	Normal to slight decrease*	Increase	Age, Gender, VAT	Respiratory motion artifacts; heterogeneous fat deposition	**High** (Widely validated biomarker)
Diffusion-Weighted Imaging & Derivatives (DKI, IVIM)	Pancreatic Cell density & membrane integrity	Lower ADC values	Lower Dt values	Age, Iron overload	Low SNR in high b-values; complex non-Gaussian models	**Moderate** (Requires specialized post-processing)
Dynamic Contrast-Enhanced MRI (DCE-MRI)	Microvascular perfusion	Unknown/Under-reported	Decrease	VAT	Requires contrast agent; demanding temporal resolution	**Moderate** (Limited by invasive contrast use)
Arterial Spin Labeling (ASL)	Non-contrast perfusion	Unknown/Under-reported	Decrease	Age, Blood flow velocity	Low intrinsic SNR; sensitive to motion and long transit time	**Low-Moderate** (Mainly used in research settings)
Magnetic Resonance Elastography (MRE)	Parenchymal stiffness/Fibrosis	Increase	Increase	Age, Body mass index	Requires external driver hardware; limited pancreatic acoustic window	**Low-Moderate** (Technically demanding for small organs)

* Quantitative CSI evaluation of pancreatic fat fraction (PDFF) in T1DM remains severely under-reported. Current evidence indicates that pancreatic steatosis is not a feature of T1DM; PDFF values in long-standing T1DM patients typically show normal to slightly decreased levels compared to healthy controls, contrasting with the prominent fatty deposition in T2DM (38, 39).

ADC, apparent diffusion coefficient; Dt, true diffusion coefficient; VAT, visceral adiposity; CSI, chemical shift imaging; PDFF, proton density fat fraction; SNR, signal-to-noise ratio.

Bold values in the “Clinical preparedness level” column (High, Moderate, Low-Moderate) indicate the grading of the clinical translation readiness or maturity level for each MRI technique.

To propel the field forward, the establishment of standardized image acquisition and post-processing protocols is imperative. Such harmonization will facilitate cross-platform comparisons and the execution of large-scale, multicenter clinical trials. The ultimate objective is the implementation of a comprehensive ‘one-stop’ MRI protocol capable of the simultaneous quantification of volumetric, steatotic, diffusive, elastic, and hemodynamic parameters. Integrating these multiparametric data will enable the construction of robust, holistic ‘pancreatic digital biomarkers.’ In the future, early screening of high-risk cohorts using qMRI could potentially attenuate the incidence of DM and substantially enhance patient outcomes. Furthermore, qMRI is poised to become a vital tool for assessing the pharmacodynamic efficacy of novel glucose-lowering agents and the potential restitution of pancreatic function.

Crucially, a pivotal milestone for clinical adoption lies in establishing the incremental added value of pancreatic qMRI over conventional biochemical surrogates, such as HbA1c, fasting glucose, and insulin resistance indices. While serum biomarkers reflect aggregate systemic metabolic states, they remain blind to localized tissue topography and the non-synchronous exhaustion of the exocrine versus endocrine pancreas. Multiparametric qMRI circumvents this diagnostic blind spot by serving as a spatially resolved ‘virtual biopsy,’ capable of detecting subclinical parenchymal remodeling, microvascular perfusion deficits, and early fibrosis long before peripheral anomalies cross clinical thresholds. Future trials must prioritize longitudinal cost-benefit analyses to demonstrate how integrating qMRI phenotypes into established clinical stratifications can fundamentally refine therapeutic decision-making.

Despite this transformative potential, a cautious stance must be maintained regarding the immediate, widespread clinical translation of multiparametric qMRI and AI-driven workflows. These cutting-edge techniques cannot blindly bypass rigorous clinical vetting; persistent hurdles in scan time, inter-vendor software discrepancies, and the lack of automated, robust segmentation models continue to impede immediate routine deployment. Consequently, the immediate roadmap for the imaging community must prioritize large-scale, prospective validation studies and rigorous multi-center standardization efforts to harmonize acquisition physics and post-processing algorithms. By positioning these standardized quantitative metrics alongside advanced radiomics and AI, future researchers will be uniquely equipped to capture the full spectrum of non-synchronous macroscopic and microscopic transformations within the diabetic pancreas, ultimately evolving qMRI from an innovative research modality into an indispensable tool for precision diabetology.
